# The role of lipid particle-laden interfaces in regulating the co-delivery of two hydrophobic actives from o/w emulsions

**DOI:** 10.1080/10717544.2024.2425158

**Published:** 2024-11-08

**Authors:** Georgia I. Sakellari, Hannah Batchelor, Fotis Spyropoulos

**Affiliations:** a School of Chemical Engineering, University of Birmingham, Birmingham, UK; b Strathclyde Institute of Pharmacy and Biomedical Sciences, University of Strathclyde, Glasgow, UK

**Keywords:** Solid lipid nanoparticles, nanostructured lipid carriers, Pickering emulsions, co-encapsulation, co-release, interfacial sintering

## Abstract

Co-delivery strategies have become an integral active delivery approach, although understanding of how the microstructural characteristics could be deployed to achieve independently regulated active co-delivery profiles, is still an area at its infancy. Herein, the capacity to provide such control was explored by utilizing Pickering emulsions stabilized by lipid particles, namely solid lipid nanoparticles (SLNs) and nanostructured lipid carriers (NLCs). These dual functional species, regarding their concurrent Pickering stabilization and active carrying/delivery capabilities, were formulated with different solid lipid and surfactant types, and the effect on the release and co-release modulation of two hydrophobic actives separately encapsulated within the lipid particles themselves and within the emulsion droplets was investigated. Disparities between the release profiles from the particles in aqueous dispersions or at an emulsion interface, were related to the specific lipid matrix composition. Particles composed of lipids with higher oil phase compatibility of the emulsion droplets were shown to exert less control over their release regulation ability, as were particles in the presence of surfactant micelles in the continuous phase. Irrespective of their formulation characteristics, all particles provided a level of active release control from within the emulsion droplets, which was dependant on the permeability of the formed interfacial layer. Specifically, use of a bulkier particle surfactant or particle sintering at the droplet interface resulted in more sustained droplet release rates. Compared to sole release, the co-release performance remained unaffected by the co-existence of the two hydrophobic actives with the co-release behavior persisting over a storage period of 1 month.

## GRAPHICAL ABSTRACT

**Figure:**
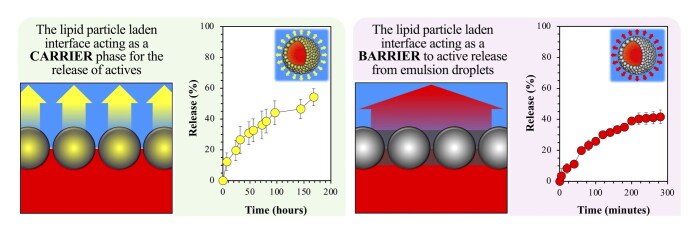

## 1. Introduction

Within the encapsulation and release research area, a range of different approaches have been explored to allow, facilitate and control the co-encapsulation and co-delivery of multiple actives. At the same time, the emergence of novel techniques to prepare such functional systems that can expedite controlled and targeted active delivery has been generating exciting possibilities for the optimization of such complex structures with diverse characteristics (Shrimal et al., 2020; Zhao et al., 2022a). Majority of the published work stems from and pertains to pharmaceuticals (Kolishetti et al., 2010; Meng et al., 2016; Kim et al., 2019; Motevalli et al., 2019), although co-delivery formulations have also found applications in agrochemicals (Cui et al., 2020; Graily Moradi et al., 2019), cosmetics (Han et al., 2020) and food products (Wei et al., 2020; Chawda et al., 2017). Incorporating more than one active ingredient within the same formulation platform has been shown to enhance efficacy and bioactivity through synergistic or additive effects between the encased species (Tavano et al., 2014; Liu et al., 2019). To this end, solid-phase formulations have been primarily investigated (Lee et al., 2019; Wang et al., 2019; Awad et al., 2019), with less attention given to liquid-based systems, despite the associated advantages that the latter can provide; in the pharmaceuticals space, these would include dosage flexibility, rapid absorption and improved patient compliance (particularly for patients unable to swallow solids) (Helal et al., 2019; Čejková & Štêpánek, 2013; Li et al., 2016).

Among the approaches utilized for multi-delivery from liquid formulations, emulsions and specifically Pickering emulsions have emerged as attractive co-delivery vehicles, due to their capacity to bestow compartmentalized encapsulation and independently controlled delivery of the incorporated actives, as well as improved emulsion stabilization. Particulates of varying physicochemical characteristics have been explored, with most studies primarily focusing on confirming the concurrent stabilization and co-encapsulation aptitude of the systems. Sun *et al*. (Sun et al., 2022) studied the co-encapsulation of three active ingredients with different solubilities, namely vitamin B_2_, vitamin E and *β*-carotene within liposome-stabilized emulsions, highlighting the impact of the microstructural components’ composition on the obtained encapsulation efficiency. With regard to the effect of co-encapsulation on the system’s properties, Chen et al. (2022) demonstrated that no obvious changes are inflicted to the size distribution of the blank emulsion, and overall improved stability following the co-incorporation of chlorogenic acid and *β*-carotene in shrimp ferritin nanocage-stabilized emulsions. In another work by Spyropoulos et al. (2018), the influence of the particles’ characteristics on attaining independent and triggered co-delivery of a hydrophobic/hydrophilic active combination from emulsions stabilized by sodium caseinate/chitosan co-precipitated complexes was highlighted. It was shown that the hydrophilic model active encapsulated within the particles exhibited a (pH) triggered-release behavior, compared to the sustained discharge of the hydrophobic active encased within the emulsion droplets.

However, the crucial role of particle-laden interfaces on the co-release performance of such Pickering emulsion-based co-delivery systems is practically unexplored in current literature. Han et al. (2021) reported that the encapsulation of a secondary active (quercetin) within black bean protein-based nanocomplexes led to more sustained release rate of the (primary) active (perilla oil) contained within the Pickering emulsion droplets, due to the creation of thicker interfacial coating, compared to blank particles. In another study, the utilization of solid lipid nanoparticles (SLNs) fabricated with a bulkier protein as the surface active species resulted in minimal release from the particles, while creation of a less permeable interfacial layer due to sintering of the SLNs led to negligible discharge of a secondary active incorporated within the oil droplets (Sakellari et al., 2021b). In a slightly different iteration of the same principle, the potential to control the active release from w/o emulsions by manipulating the architecture of their lipid-decorated interfaces via sintering, was shown to lead to triggered release, through temperature control (Garrec et al., 2012; Frasch-Melnik et al., 2010). However, such an approach whereby the production of Pickering entities is fine-tuned prior to emulsion formation and their sintering is attuned *in-situ* at the droplet interface to achieve bespoke active release performances, is yet to be explored within a co-delivery setting.

The current work aimed to investigate the role of lipid particle-laden emulsion interfaces in terms of regulating the co-delivery performance of Pickering o/w emulsions (Sakellari et al., 2022, 2023). The effect of formulation characteristics of the lipid particles, both SLNs and nanostructured lipid carriers (NLCs), on their simultaneous emulsion stabilization and active carrying/release regulation capabilities were studied. The release profiles of a model hydrophobic active (curcumin) encapsulated within SLNs and NLCs of varying formulations aspects, namely the types of solid lipid and surfactant used (during particle fabrication), were assessed in two settings; firstly, when the particles are simply dispersed in an aqueous medium (aqueous dispersions) and secondly, when the particles are (predominantly) positioned at an emulsion interface. Focus was also placed on examining the capacity and extent to which (blank) lipid particle interfaces could act as effective interfacial barriers and provide control over the encasing and release of a secondary model hydrophobic active (cinnamaldehyde) encapsulated within the (lipid particle-stabilized) o/w emulsion droplets; release behavior in this case was compared to that of simple (surfactant-stabilized) emulsions. Interfacial sintering was devised as a means to manipulate the interfacial barrier provided by the lipid particles and scrutinize how and to what degree this could provide further control over the release of the secondary active. Finally, the co-release of both model hydrophobic actives from SLN and NLC particle-stabilized emulsions was studied to confirm the co-encapsulation and co-delivery functionality of the developed formulation.

## 2. Materials and methods

### 2.1. Materials

Compritol^®^ 888 ATO (C888, glyceryl behenate) and Precirol^®^ ATO 5 (P5, glyceryl palmitostearate) were kindly provided from Gattefossé (Saint-Priest, France). Miglyol^®^ 812 (medium chain triglycerides, MCTs) was a kind gift from IOI Oleo (IOI Oleochemicals GmbH, Germany). Tween^®^ 80 (T80, polyoxyethylene sorbitan monooleate), Pluronic^®^ F-68 (Poloxamer 188, P188), curcumin (≥65%, HPLC, CRM), pentane (HPLC grade) and cinnamaldehyde (CA) were purchased from Sigma–Aldrich (Sigma–Aldrich, UK). Oxoid^™^ phosphate-buffered saline (PBS) pH 7.4 tablets were obtained from Thermo Scientific (Sheffield, UK). Sunflower oil was purchased from a local supermarket, stored in a closed container at ambient temperature in the dark, and used without any further purification. Double distilled water from Milli-Q systems (Millipore, Watford, UK) was used during all sample preparation processes and characterization measurements.

### 2.2. Preparation of lipid particles

The aqueous dispersions of blank or curcumin-loaded SLNs and NLCs were prepared following a melt-emulsification-ultrasonication method that is fully described elsewhere (Sakellari et al., 2021a). The lipid melts (2.5% w/w) without or with curcumin (0.5% w/w of the lipid mass) were heated 5–10 °C above the melting point of the solid lipid used (85 °C for C888 and 70 °C for P5) for 1 hr and were then combined with the aqueous surfactant solution (1.2% w/w T80 or P188). For the NLCs, 30% of the total lipid phase was substituted by MCTs. The formed pre-emulsion was homogenized for 5 min using ultrasonication (Vibra-cell^™^ VC 505 Processor, Sonics & Materials, Inc., CT, USA), operating continuously at 750 Watt and 20 kHz, at a sonication amplitude of 95% of the total power. The crystalline particles were obtained by cooling the o/w emulsion using an ice bath to a temperature below the crystallization point of the lipid melts. Samples were stored at 4 °C in the dark (since curcumin is photodegradable) until further analysis.

### 2.3. Particle size

Information about the particle size (*Ζ*-average) and polydispersity index (PDI) of the SLNs/NLCs was acquired with dynamic light scattering (DLS), using Zetasizer Nano ZS (Malvern Instruments, UK). All measurements were performed at a backscattering angle of 173° at 25 °C, and samples were appropriately diluted with distilled water to avoid multiple scattering phenomena. The refractive indices were determined according to Sakellari et al. (2021a, 2022). All measurements were performed in triplicate, immediately after preparation and over time, and the average values with standard deviation (±S.D.) are presented. Representative size distributions of lipid particles were also obtained with laser diffraction (LD) using a Mastersizer 2000 (Malvern Instruments, UK), following a method that is described in detail below.

### 2.4. Interfacial tension

Dynamic interfacial oil/water tensions at 20 °C were measured with a profile analysis tensiometer, using the pendant drop method (PAT-1M, Sinterface Technologies, Berlin, German). A drop of the SLN or NLC dispersions was suspended via a straight stainless-steel capillary (3 mm outer diameter) in the sunflower oil phase contained in a quartz cuvette, with the cross-sectioned surface area remaining constant at 27 mm^2^. The measurements were performed until equilibrium was reached (the standard deviation of the last twenty measurements was smaller than 0.05 mN/m). Density information was acquired using a densitometer (Densito, Mettler Toledo, US), at 20 °C. All measurements were conducted in at least triplicate on three individually prepared samples.

### 2.5. Preparation of oil-in-water emulsions

Simple or Pickering o/w emulsions were prepared with 90% (w/w) aqueous phase containing either of the two surfactants at 1% w/w concentration, or any of the different (blank or curcumin-loaded) lipid nanoparticle systems, respectively, and 10% (w/w) sunflower oil phase. When cinnamaldehyde (0.3% w/w of the oil phase mass) was encapsulated within the oil droplets, the active and sunflower oil were stirred together for 1 h prior to the aqueous phase addition. During emulsification, which was performed employing ultrasonication under the same conditions as described above for a period of 30 s, the samples were immersed in an ice bath to avoid shear-inducing heating and were later stored at 4 °C until further analysis.

Emulsions that were thermally processed post-fabrication were heated at either 64 or 78 °C (according to the melting events observed in the thermograms of the particle-stabilized emulsions) for 5, 20 or 60 min (after the desired temperature was reached) using a hotplate under stirring, and were then cooled in an ice bath.

### 2.6. Droplet size measurements

Laser diffraction (LD) was utilized to obtain droplet size information, employing a Mastersizer 2000 (Malvern Instruments, UK) equipped with a Hydro SM manual small-volume sample dispersion unit. The stirrer speed was set at 1300 rpm, and all samples were hand-mixed before analysis. The refractive index for sunflower oil was set at 1.47. All measurements were performed in triplicate on three individually prepared samples.

### 2.7. Thermal analysis

The thermal behavior of the SLNs and NLCs within the dispersion and emulsion systems was evaluated via Differential Scanning Calorimetry (DSC) using a Setaram μDSC3 evo microcalorimeter (Setaram Instrumentation, France). The temperature cycle used ranged between 20 and 80 °C at a heating rate of 1.2 °C/min. The thermograms were obtained with the reference cell being filled with equal amount of distilled water. Data processing was carried out using the Calisto Processing software (Setaram Instrumentation, France), to obtain information regarding peak temperatures and melting enthalpies. The loss of crystalline matter for the emulsion systems was determined using information from the total melting enthalpies of the particles within an emulsion environment (Δ*H^T^_em_*) and those in an aqueous dispersion setting (Δ*H_dis_*) and was expressed as a Δ*H^T^_em_*/Δ*H_dis_* ratio. The Δ*H_dis_* and Δ*H^T^_em_* values were obtained from peak integration of the particle dispersion and particle-stabilized emulsion melting thermograms, respectively. All enthalpy values and thermograms reported, are normalized for the crystallizing material amount present in each sample. Specifically for the emulsion systems, each thermogram was normalized using the information of the respective SLN or NLC dispersion that was used for the emulsification. All measurements were performed in at least duplicate.

### 2.8. Encapsulation efficiency and loading capacity

The encapsulation efficiency (EE) and loading capacity (LC) of CRM-loaded particles and CA-loaded droplets was assessed by ultrafiltration using centrifugal ultrafiltration tubes (Amicon^®^ Ultra-4 filter 10 kDa cutoff, Millipore, Billerica, MA, USA). 1 mL of either the dispersion or emulsion systems was added to the upper chamber of the centrifugal tube and centrifuged at 2,400 rcf for 1 h at room temperature using a SIGMA 3K-30 centrifuge (SciQuip^®^, UK). The concentration of unentrapped CRM or CA in the filtrate was subsequently determined by measuring the UV–Vis absorbance (Genova Bio Life Science Spectrophotometer, Jenway^®^, Cole-Palmer, UK) at 425 or 278 nm, respectively. Explicitly for CA, a solvent extraction protocol was followed prior to the absorbance measurements to eliminate any co-absorption interference at the specific wavelength. An aliquot of the filtrate was mixed with pentane at a 1:2 ratio, and the CA-rich pentane phase was then measured to determine the absorbance. The concentration of each model active was determined using calibration curves previously generated, with linearity studied for 0–6 μg/mL and linear regression value of *R*^2^ = 0.9995 for CRM, and linearity of 0–28.7 μg/mL and *R*^2^ = 0.9915 for CA. The EE and LC values were calculated using the following equations:

(1) $$\text{EE}=\frac{W_{i.}-W_{u.}}{W_{i.}}\times 100(\%)$$

(2) $$\text{LC}=\frac{W_{i.}-{\;W}_{u.}}{W_{l.p.}}\times 100(\%)$$

where *W_i._* is the amount of active that was initially used during the preparation of the aqueous lipid dispersions or emulsions, *W_u._* is the amount of active measured in the filtrate, and *W_l.p._* is the total amount of the lipid/oil components used in the systems.

### 2.9. In vitro release and co-release

*In vitro* release of curcumin, from curcumin-loaded lipid particle dispersions and particle-stabilized (Pickering) o/w emulsions was performed by diffusion through a dialysis membrane. A known amount of the particle dispersions or the Pickering emulsions was enclosed in a cellulose dialysis membrane (43 mm width, 14 kDa M.W. cutoff, Sigma-Aldrich Company Ltd., Dorset, UK), and the tubing was introduced in the *in vitro* release medium (130 g) consisting of phosphate buffer saline (PBS, pH 7.4) and 1.0% w/w Tween^®^ 80. At predetermined time intervals, 1 mL aliquots of the dissolution medium were collected and analyzed by UV–Vis spectrophotometry (Genova Bio Life Science Spectrophotometer, Jenway^®^, Cole-Palmer, UK). The *in vitro* release of cinnamaldehyde-loaded simple or Pickering emulsion droplets was assessed following the exact same protocol, with a slight modification at the absorbance measurement step, accordingly to what was previously described for the EE and LC determination. The absorbance was measured at 425 nm for CRM and at 278 nm for CA. The release measurements were performed using an Incu-Shake MIDI shaker incubator (Sciquip, UK) operating at 25 °C under constant shaking (180 rpm). The dissolution of CRM and CA solutions (at equal concentrations as those used in the dispersion/emulsion systems) prepared using the dissolution medium as solvent was also assessed. The dialysis membranes were soaked in the dissolution medium overnight, prior to usage. Sink conditions were maintained by replacing the sampled aliquots with equal volume of fresh media. The volume correction has been accounted for, in the reported cumulative release plots. The measurements were conducted in triplicate using independently prepared samples.

For the co-release assessments, the only adaptation in the above-described method was relevant to doubling the volume of aliquots withdrawn, to allow for sufficient quantification volume. For the stability assessments, the co-release measurements were performed immediately after preparation and after 1 month of storage at 4 °C.

### 2.10. Modeling of release data

The release data from the CRM-loaded lipid particles either in dispersion or emulsion settings were fitted into the mechanistic model described by Crank (1975), to gain further insight regarding the underlying release mechanism. The diffusion coefficient (*D*) was determined as follows:

(3) $$\frac{Q_{t}}{Q_{\infty }}=1-\frac{6}{\pi^{2}}\sum_{n=1}^{\infty }\frac{1}{n^{2}}e\text{xp}(-\frac{Dn^{2}\pi^{2}t}{r^{2}})$$

where *Q_t_* is the mass of active released at time *t*, *Q_∞_* is the total mass of active released when the formulation is exhausted, *n* is the number of the term in the series, *r* is the particle radius (calculated using the *Z*-average), and *D* is the apparent diffusion coefficient of the active within the system.

Regarding the release of actives from within emulsion droplets, two limiting models have been previously described (Guy et al., 1982; Washington & Evans, 1995) and utilized in literature (Kurukji et al., 2016; Spyropoulos et al., 2020; Sakellari et al., 2021b). According to these, the release of the active is either primarily driven by diffusion through the oil droplet, or it is limited by the presence of an interfacial barrier around the emulsion droplet.

When the former is true, there is no interfacial barrier effect on the diffusion of the active through the oil core, and the release at long times can be approximated by:

(4) $$\text{ln}\;(1-\frac{Q_{t}}{Q_{\infty }})=\text{ln}\;(\frac{6}{\pi^{2}})-\frac{\pi^{2}D}{r^{2}}t$$

which is the linear form of the following:

(5) $$\frac{Q_{t}}{Q_{\infty }}=1-\frac{6}{\pi^{2}}e\text{xp}(-\frac{\pi^{2}D}{r^{2}}t)$$

where symbols retain their previous meaning (Eq. (3)), but relevant to the emulsion droplets. For the emulsion radius (*r*), the *D_3,2_* data acquired from the LD measurements was used. Using Eq. (4) and plotting the *ln*(1−*Q_t_/Q_∞_*) against time will have a limiting slope of (*π*^2^*D/r*^2^), which can be used to calculate the diffusion coefficient *D*.

For the alternative model, where the active discharge is governed by the transfer across the interfacial barrier and the active is considered to be uniformly distributed within the emulsion droplet at all times, the following long-time approximation equation was used:

(6) $$\frac{r^{2}}{3}\text{ln}(1-\frac{Q_{t}}{Q_{\infty }})={-k}_{1}t$$

which is the linear form of the following:

(7) $$\frac{Q_{t}}{Q_{\infty }}=1‐\text{exp}(\frac{-3k_{1}}{r^{2}}t)$$

where *k*_1_ is the interfacial rate constant, with all other symbols retaining their meaning. Comparably to the previous model, plotting the natural logarithmic term on the left-hand side of Eq. (6) against time would provide with a straight line, the slope of which can be used to calculate *k*_1_.

The *D* values attained here were in the range of 10^−11^, and thus significantly lower than those calculated using the Stokes–Einstein equation (*D*** **=** **1.3 × 10^−7^ cm^2^ s^−1^) for a small molecule diffusing through sunflower oil. If release was driven by diffusion, it would be expected that the estimated values would be close to that calculated by the Stokes–Einstein equation, but also close to one another, as the same oil phase was utilized in all formulations (Washington & Evans, 1995; Kurukji et al., 2016; Spyropoulos et al., 2020; Sakellari et al., 2021b). Therefore, the release should be primarily governed by transfer across the interfacial layer, rather than diffusion, and any *k*_1_ differences should come as a result of interfaces with different barrier characteristics. The interfacial barrier-limiting model was further considered for the emulsion release behavior at longer times (*t*** **≥** **30 min).

### 2.11. Statistical analysis

Samples were analyzed in at least triplicate, and averages are reported with standard deviation. Figures depict the calculated average value with error bars showing the standard deviation above and below the average. Comparison of means was conducted by ANOVA analysis followed by an all-pairwise multiple comparison test using the Student-Newman-Keuls Method (SigmaPlot 14.5). The differences were considered statistically significant when *p*** ***≤*** **0.05.

## 3. Results and discussion

### 3.1. Release from lipid particles

Recently, SLNs and NLCs were utilized as dual functional species, with the intent of simultaneously regulating the encapsulation/release of curcumin (used model hydrophobic active) and acting as Pickering emulsion stabilizers (Sakellari et al., 2022, 2023). Though the introduction of the loaded particles within the emulsion system was shown to accelerate the release rate compared to that recorded in a dispersion setting, both types of particles were still able to regulate the discharge of curcumin, with overall sustained release profiles being reported (∼50% released over 7 days), while maintaining their Pickering stabilization capacity (Sakellari et al., 2023). Herein, the impact of modifying lipid particle formulation aspects, namely the type of solid lipid used and its combination with a liquid lipid, as well as the type of surfactant employed, on the release behavior in both an aqueous dispersion and an emulsion setting are explored.

#### 3.1.1. Lipid particles in aqueous dispersions

**Figure 1.**
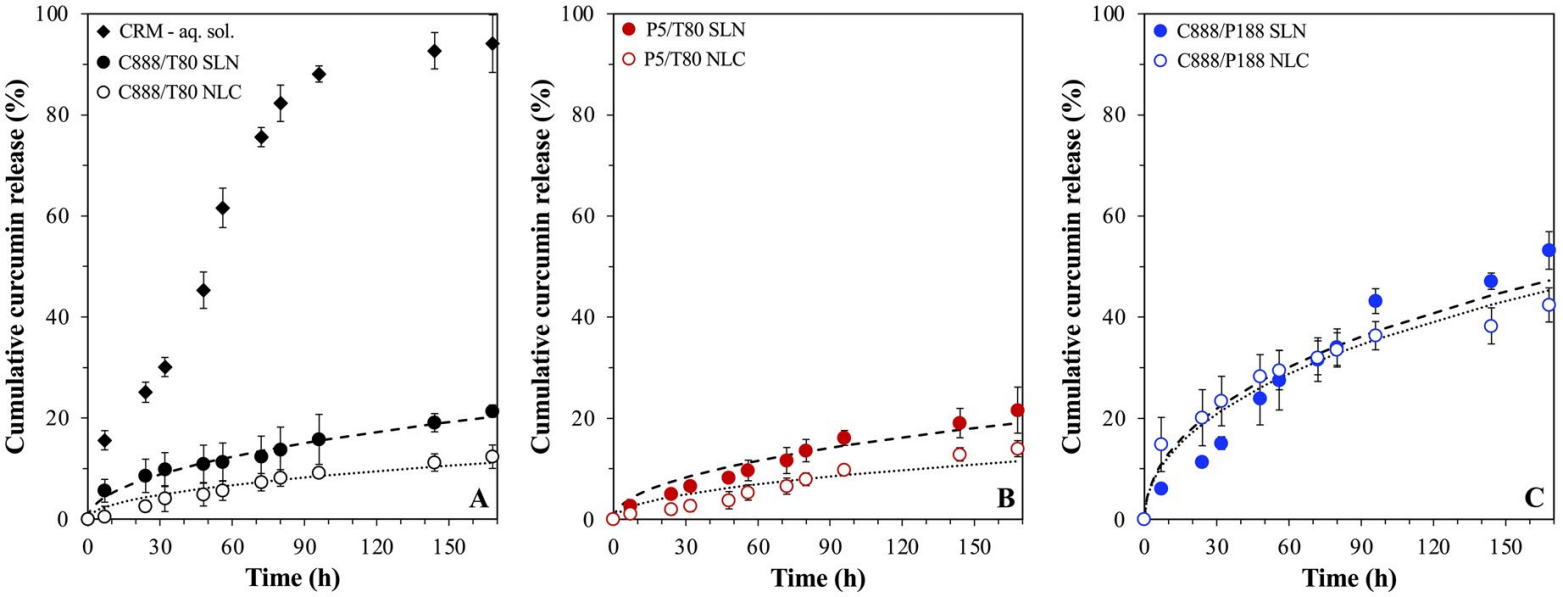
*In vitro* release profile of curcumin-loaded SLN and NLC dispersions formulated with either Tween^®^ 80 (T80) as surfactant and different types of solid lipid (A) Compritol^®^ 888 ATO (C888) and (B) Precirol^®^ ATO 5 (P5), or different surface active species (C) Poloxamer 188 (P188) and C888 as solid lipid. NLCs were fabricated with Miglyol^®^ 812 as the liquid lipid, at 30% w/w of the total lipid phase mass. The release profile from a curcumin solution obtained under the same conditions is also depicted (a). The *in vitro* release kinetic Crank model (Eq. (3)) fitting of curcumin for each SLN (dashed line) and NLC (dotted line) dispersion is also presented. Graph A has been previously shown in (Sakellari et al., 2023) and is provided here for comparison purposes.

Prior to introducing the lipid particles within the emulsion systems, their performance in aqueous dispersions was explored to establish how changes to the formulation parameters affect their release regulation ability (Figure 1). Based on previous investigations of the release performance of SLNs and NLCs fabricated with C888 as the solid lipid and MCTs as the liquid lipid, it was proposed that addition of MCTs results in the structural reorganisation of the particles’ lipid matrix into a less ordered crystalline state, a phenomenon that in turn decreased the rate of curcumin release (Sakellari et al., 2023). Additionally, the release mechanism was described as diffusion-driven, due to both the overall slow release rate and high EE/LC values for either type of particles, and the low diffusion coefficients (*D*) as determined by the Crank model (Eq. (3)).

**Figure 2.**
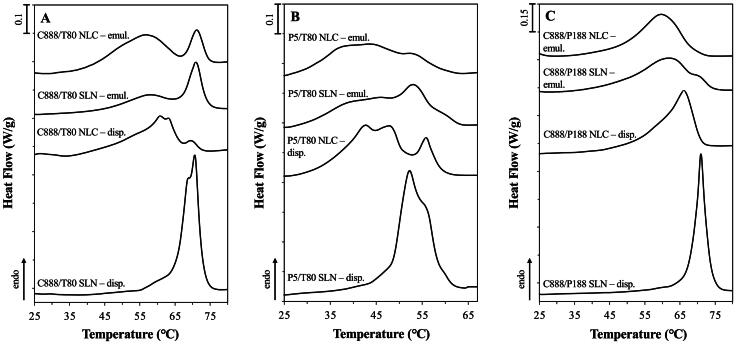
DSC melting thermograms of SLN and NLC dispersions and their respective Pickering emulsions, for lipid particles formulated with either Tween^®^ 80 (T80) as surfactant and different types of solid lipid (A) Compritol^®^ 888 ATO (C888) and (B) Precirol^®^ ATO 5 (P5), or different surface active species (C) Poloxamer 188 (P188) and C888 as solid lipid. NLCs were fabricated with Miglyol^®^ 812 as the liquid lipid, at 30% w/w of the total lipid phase mass. The curves were normalized for the amount of solid matter present in each sample and shifted along the ordinate for better visualization. Graph A has been previously shown in literature (Sakellari et al., 2022, 2023) and is provided here for comparison purposes.

Delving further into the influence of modifying the lipid particles’ composition, the high melting point solid lipid C888 was substituted by the lower melting point P5, keeping the rest of the formulation aspects unchanged, and the release performance of P5 SLNs and NLCs was investigated (Figure 1(B)). Compared to their C888 counterparts (Figure 1(A)), both lipid particle types demonstrated almost identical release profiles and very similar *D* values (Table 1). According to previously reported solubility studies (Sakellari et al., 2021a), C888 and P5 have comparable CRM solubility thresholds (0.6 and 0.7%, respectively), while their EE and LC values were the same (99.9 ± 0.0% and 0.5 ± 0.0%, respectively). Considering the above, it could be assumed that the localization of curcumin and/or internal arrangement of the lipid particles, for both the SLNs, but also the NLCs pairs were akin. Several studies have discussed the incapacity of the crystalline lipid structure of SLNs to host active molecules, and the subsequent expulsion/migration of the latter toward the surface of the particles (Jores et al., 2004; Kishore et al., 2012; Gordillo-Galeano et al., 2022). Correspondingly, addition of the liquid lipid component within the solid matrix has been associated with solid/liquid phase separation, particularly at higher liquid lipid concentrations, and concentration-dependent creation of distinct lipid structures (Jenning et al., 2000; Jores et al., 2003; Sakellari et al., 2021a). With reference to the latter, it has been reported that once the solid lipids’ solubility limit for the liquid component is exceeded, liquid oil nano-compartments can be formed within the matrix, or the liquid lipid can be concurrently expelled toward the surface (Jores et al., 2004, 2005). The formation of these compartments requires that sufficient space is available within the arrangement/packing of the crystalline element. Specifically for C888, when the lipid is crystallized at a cooling rate of 1 °C/min, which is very close to the one used in this work (1.2 °C/min), the co-presence of two lamellae has been reported (Brubach et al., 2007), leading to matrix imperfections (Souto et al., 2006). Recently, Gordillo-Galeano et al. (2022) described the stages of active (paraben) exclusion as the lipid matrix crystallizes, toward the MCTs-rich surface of trimyristin NLCs, or in-between the trimyristin crystals and the surfactant (P188) layer in SLNs. In the present study, both C888 and P5 are mixtures of variable triacylglycerols; the former of slightly longer alkyl chains, while the latter of more varied composition (behenic acid and palmitic/stearic acid esters, respectively). Additionally, in both types of NLCs, the prospect of the presence of polymorphs characterized by lower packing densities has been previously discussed (Figure 2(A and B)) (Sakellari et al., 2021a), thereby not utterly excluding the possibility of MCTs compartment formation within the NLCs’ structure (Macridachis-González et al., 2020; Bertoni et al., 2021). However, taking into account that MCTs was used at a 30% w/w concentration of the total lipid phase, which was likely exceeding the solubility limits of either solid lipids for MCTs, it was expected that the bulk of the liquid lipid component would be contained near the surface of the particles (Jores et al., 2004, 2005). Consequently, given the solubility constrains imposed by the solid lipid crystallization, it could be hypothesized that CRM would be preferentially located within the MCTs phase (NLCs) or near the surface of the particles (SLNs), at least when it comes to its highest proportion (Anantachaisilp et al., 2010; Shah et al., 2012; Gordillo-Galeano et al., 2022).

**Table 1.** Diffusion coefficient (*D*) and coefficient of determination (*R*^2^) describing the fitting into the Crank model (Eq. (3)) of the curcumin release data from lipid particles within dispersion and emulsion systems. Identical lowercase letters indicate no significant differences between samples.

Lipid particle setting	Lipid formulation	*D*** **×** **10^−20^ (cm^2^s^-1^)	*R* ^2^
	C888/T80 SLN	45.1 ± 7.9^a^	0.98
Dispersions	C888/T80 NLC	12.1 ± 1.4^a^	0.97
	P5/T80 SLN	45.0 ± 3.7^a^	0.96
	P5/T80 NLC	6.6 ± 1.2^a^	0.95
	C888/P188 SLN	214 ± 34.5^b^	0.94
	C888/P188 NLC	178 ± 23.1^b^	0.98
Emulsions	C888/T80 SLN	213 ± 23.2^b^	0.94
	C888/T80 NLC	177 ± 25.4^b^	0.97
	P5/T80 SLN	546 ± 45.1^c^	0.96
	P5/T80 NLC	171 ± 45.8^b^	0.98
	C888/P188 SLN	278 ± 39.0^b^	0.99
	C888/P188 NLC	310 ± 51.0^b^	0.99

The next parameter assessed was the effect of lipid particle formation in the presence of different surface active species, on the CRM release, with both SLNs and NLCs fabricated with P188 as surfactant and C888 as the solid lipid (Figure 1(C)). Compared to their T80 counterparts, both P188 particle types exhibited faster release. Furthermore, there was almost no difference between the C888/P188 SLN and NLC formulations, as opposed to the slower NLC release recorded for the particles fabricated with T80 for either type of solid lipid. The *D* values calculated for these systems (Table 1), although higher than the particles formed with T80, were still significantly lower than values reported in literature for curcumin-loaded SLNs fabricated with stearic acid as the lipid phase and P188 as surfactant (Tiyaboonchai et al., 2007; Sakellari et al., 2021b). The Crank model described well the diffusion-driven release, despite the slightly less ideal fitting for C888/P188 SLNs, which could be explained by the initially predicted faster release by the model, compared to the experimentally collected data. Taking into consideration that there were only very small particle size differences (Table 2), and the EE/LC values remained the same in all particle types (Sakellari et al., 2021a), it could be suggested that the discrepancies in the release behavior were driven by the surfactant substitution. Based on the melting behavior of the C888/P188 lipid particles where a diminished degree of polymorphism was observed (Figure 2(C)), and previous investigations regarding the compatibility between the lipid matrix components (Sakellari et al., 2021a), T80 appeared to participate at a greater extent (than P188) within the crystalline network (close to the particle surface). Therefore, it could be also postulated that the co-existence of C888/T80 in SLNs or MCTs/T80 in the case of NLCs at the edge of the particles’ structure and near the particles’ surface is posing an additional barrier for CRM to cross, possibly due to the creation of a favorable hydrophobic environment compared to the dissolution medium. Contrary to the potentially denser packing arrangement provided by the smaller T80 molecules, the larger molecular sized P188 molecules could not provide an as tightly packed interface, which together with their limited crystalline network participation, due to their more hydrophilic character, could not permit a strong retention of CRM, in both the P188-decorated SLNs and NLCs (Badawi et al., 2020).

**Table 2.** *Z*-average and polydispersity index (PDI) of different SLN and NLC formulations measured after preparation.

Formulation	*Z*-average (nm)	PDI
C888/T80 SLN	165.1 ± 2.7	0.20 ± 0.02
C888/T80 NLC	163.2 ± 3.8	0.12 ± 0.01
P5/T80 SLN	176.0 ± 9.6	0.28 ± 0.04
P5/T80 NLC	134.4 ± 7.1	0.14 ± 0.04
C888/P188 SLN	139.7 ± 1.9	0.20 ± 0.01
C888/P188 NLC	133.7 ± 2.1	0.18 ± 0.02

With respect to the sustained release rate from all six lipid particle types (<50% CRM released cumulatively in 7 days), similar trends have been previously reported for SLNs and NLCs encapsulating hydrophobic moieties (Ugazio et al., 2002; Hu et al., 2004; Venkateswarlu & Manjunath, 2004; Luo et al., 2006). Such sustained release profiles were usually ascribed to hindered diffusion of the active molecules through the highly ordered crystalline arrangement of the lipid particles, though active partition coefficients and solubility constraints could also contribute to the stunted release percentages and lengthy experimental times (Bunjes, 2010; Noack et al., 2012; Salminen et al., 2014, 2016). Herein, the dissolution medium was selected based on two criteria; firstly, it was prerequisite to have sufficient solubilization capacity for the amount of active present in the particle dispersions during the release studies (sink conditions), and secondly based on previously reported literature (Shahani & Panyam, 2011; Zhao et al., 2022b), and its use in similar systems, it was anticipated that it would not impact on the lipid particles’ integrity over the timescales of the measurement, to ensure that the effect of the formulation aspects was adequately represented. However, it should also be noted that the selection of a solvent for which CRM has a higher affinity compared to the selected lipids, and hence would result in lower partition coefficient (Zur Mühlen et al., 1998; Rosenblatt & Bunjes, 2009), and/or higher solubility thresholds, could have also potentially led to much faster release rates. Therefore, the results presented here in terms of the release profiles, but also with regard to the diffusion coefficients, should only be approached as a relative measure of the influence of formulation parameter changes.

#### 3.1.2. Lipid particles in emulsions

The dual role of both SLNs and NLCs to act as Pickering stabilizers and in tandem as release regulators of CRM has been previously shown, using particles fabricated with C888 and T80 (Sakellari et al., 2022, 2023). Particular focus was placed on the lipid particle and particle-stabilized emulsion properties that can have an effect on the release behavior (e.g. solid-to-liquid lipid mass ratio of the lipid particles), and the underlying mechanism that drives said release. It was suggested that the CRM discharge from particle-loaded Pickering emulsions is the composite of the release from particles remaining dispersed in the continuous aqueous phase, and particles positioned at the oil-water droplet interface (Sakellari et al., 2023). The increased release rate from emulsions, compared to that from particle dispersions, was attributed to partial migration of particle-entrapped curcumin to the oil phase (droplets) as well as to a limited loss of crystalline matter from the particles (again into the emulsion droplets). Therefore, before probing deeper into the release performance of lipid particles with modified formulation parameters, namely type of solid lipid and surfactant, physical characteristics of both the particle dispersions and the respective particle-stabilized emulsions were scrutinized. This was performed to gain further insight into the microstructural properties of the formed systems, particularly in terms of their Pickering behavior.

**Figure 3.**
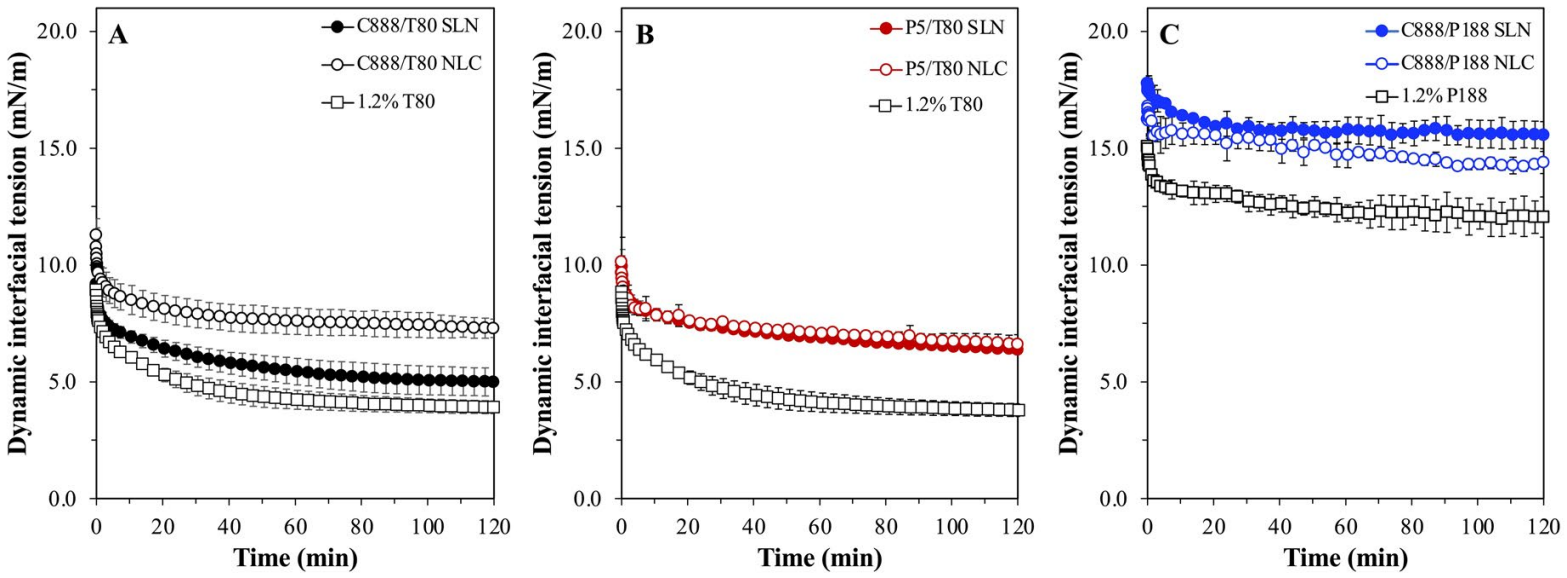
Dynamic interfacial tension of aqueous dispersions of SLN and NLC formulations prepared with either Tween^®^ 80 (T80) as surfactant and different types of solid lipid (A) Compritol^®^ 888 ATO (C888) and (B) Precirol^®^ ATO 5 (P5), or different surface active species (C) Poloxamer 188 (P188) and C888 as solid lipid. NLCs were fabricated with Miglyol^®^ 812 as the liquid lipid, at 30% w/w of the total lipid phase mass. The curves of pure T80 and P188 solutions with similar concentration (1.2% w/w) as of those used for the dispersions are also presented for comparison. Data points are the average of three measurements and error bars represent the standard deviation. Graph A has been previously shown in (Sakellari et al., 2022) and is provided here for comparison purposes.

**Figure 4.**
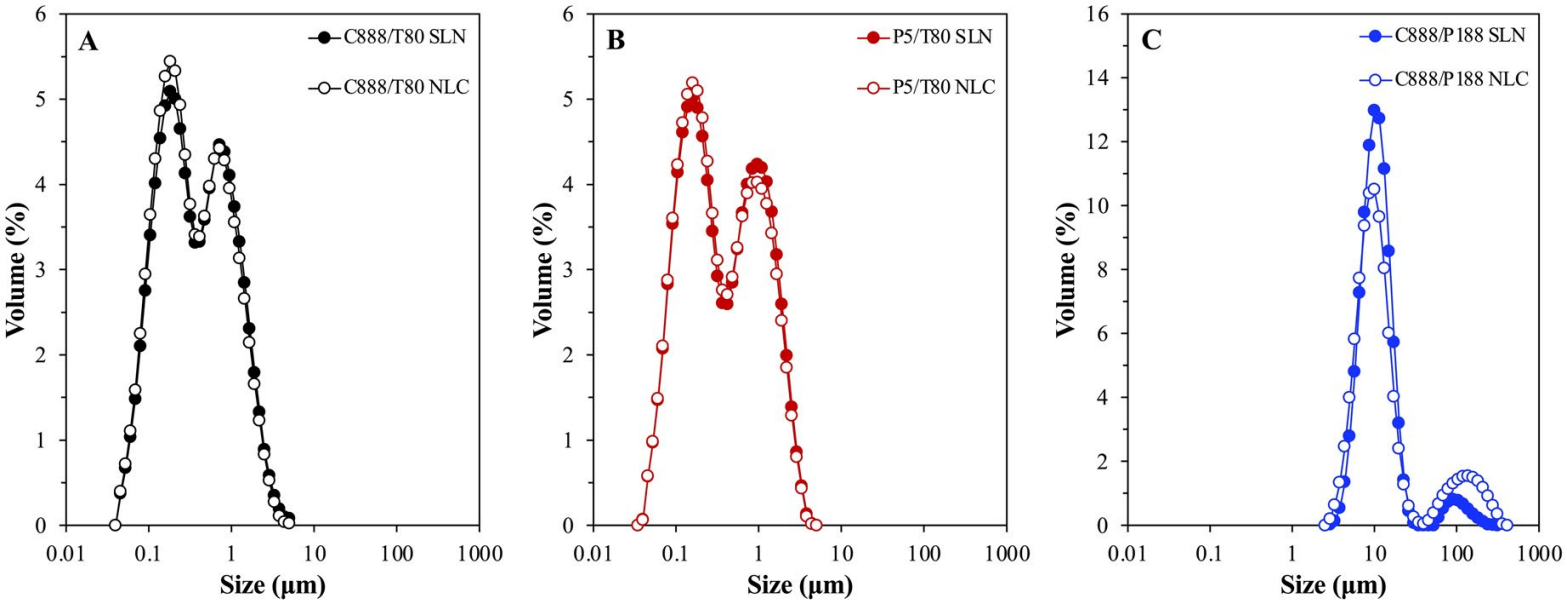
Droplet size distribution of SLN- and NLC-stabilized emulsions, with lipid particles formulated with either Tween^®^ 80 (T80) as surfactant and different types of solid lipid (A) Compritol^®^ 888 ATO (C888) and (B) Precirol^®^ ATO 5 (P5), or different surface active species (C) Poloxamer 188 (P188) and C888 as solid lipid. NLCs were fabricated with Miglyol^®^ 812 as the liquid lipid, at 30% w/w of the total lipid phase mass. Graph A has been previously shown in (Sakellari et al., 2022) and is provided here for comparison purposes.

Among these was the interfacial tension reduction capacity of the particles (Figure 3), based on which information regarding the interfacial decoration of the particles could be extracted. It was observed that both P5 fabricated particle types had identical interfacial tension reduction ability with an equilibrium value of 6.3 mN/m (Figure 3(B)), which was similar as that recorded for the C888 NLCs (Figure 3(A)), but higher than the C888 SLNs. Considering that all T80-formed particles had similar sizes, and an equal amount of surfactant was employed during their fabrication, this disparity could be attributed to differences in the lipid composition of the particles affecting the arrangement and packing density of surfactant molecules at their surface. Particles prepared with P188 as the surfactant demonstrated considerably higher equilibrium interfacial tension values; 15.3 and 14.3 mN/m for the SLNs and NLCs, respectively (Figure 3(C)). This could be due to differences in the molecular size of the two surfactants, which in turn could be resulting in different packing arrangements at the particles’ surface. T80 seemed to instigate a higher interfacial tension reduction compared to P188, possibly owing to a higher and more tightly packed interfacial presence, caused by its smaller molecular size, as supported by the trends of both aqueous surfactant solution curves (Zafeiri et al., 2017a). The effect of the surfactant molecular size was also reflected in the obtained emulsion droplet sizes. Regardless of the type of solid lipid used or type of lipid particle (SLN/NLC) created, particles fabricated with T80 exhibited almost identical droplet size distributions (Figure 4(A and B)), while P188 particles formed droplets that were an order of magnitude larger (Figure 4(C)).

**Figure 5.**
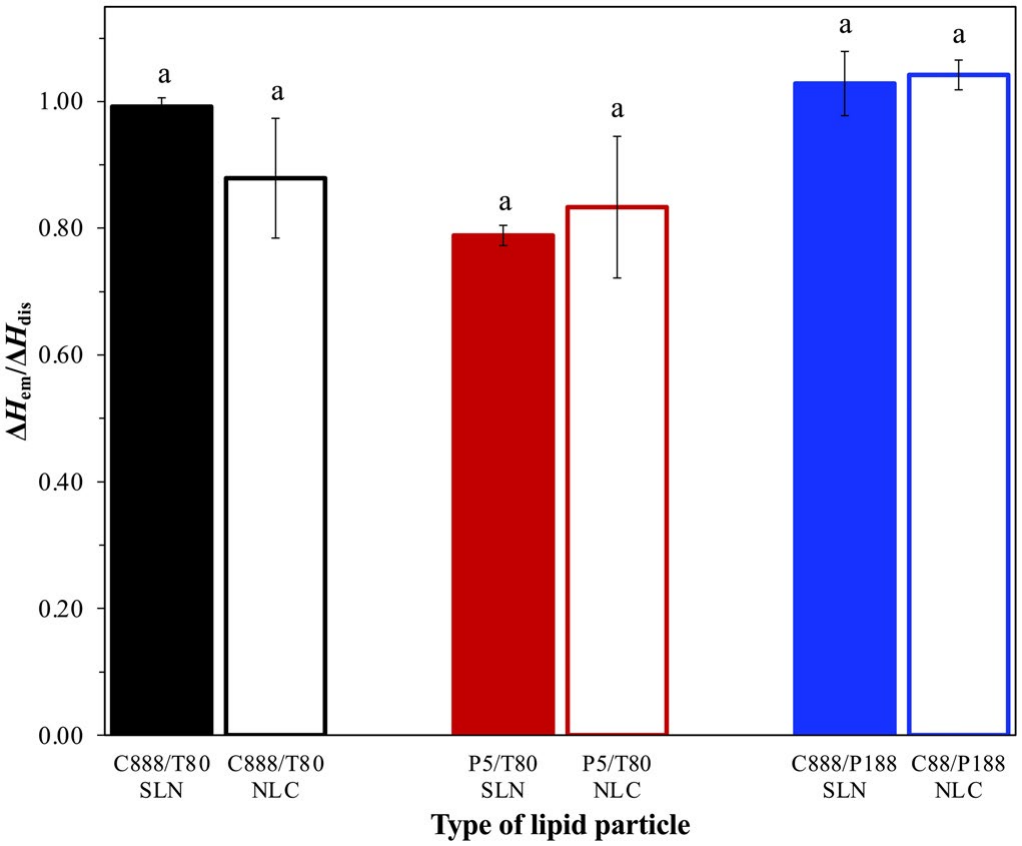
Ratio of the melting enthalpies of the particles within an emulsion environment and those in a lipid particle dispersion setting (Δ*H^T^_em_*/Δ*H_dis_*), representing the amount of crystalline material remaining within the emulsions stabilized by different types of lipid particles. Identical lowercase letters indicate no significant differences between samples.

The loss of crystalline matter once the particles were introduced within the emulsion systems was also explored, as this has been suggested as a factor impacting their release behavior. According to Figure 5, there was a greater loss of crystalline material for the P5/T80 SLNs/NLCs compared to the C888 containing particles. As it has already been suggested in literature (Samtlebe et al., 2012; Zafeiri et al., 2017b; Sakellari et al., 2023), the type of lipid source used can play a crucial role in lipid mass transfer phenomena, with surfactant micelles facilitating the transfer of oil molecules from the emulsion droplets to the particles’ lipid core. Such occurrences could either be directly related to particles adsorbed at the emulsion interface or even particles remaining free in the continuous phase. In this instance, it appeared that the potentially higher compatibility of P5 (glyceryl palmitostearate) and sunflower oil (containing triglycerides of palmitic and stearic acids), due to their closer chemical composition compared to that between C888 and sunflower oil, could be further aiding dissolution (Jamieson & Baughman, 1922; Sakellari et al., 2021a). On the contrary, emulsion formation did not cause any losses of crystalline matter for the C888/P188 particles, possibly owing to the improved protection provided by the P188 molecules over such incidents. As mentioned earlier, besides the type of lipid source, another aspect affecting this event was the formation and presence of surfactant micelles within the continuous phase (McClements et al., 1993a,b; McClements & Dungan, 1993; Weiss & McClements, 2000); in this instance, introduced to the system from the continuous phase of the particle dispersions (remnant unadsorbed surfactant). A previous study exploring the impact of removal of excess unadsorbed surfactant from the continuous phase of the lipid particle dispersions (prior to their use during emulsification) on crystalline matters loses, revealed that although there was no difference for dialyzed and undialysed SLNs, when it came to NLCs, dialysis helped decrease mass transfer phenomena following the addition of the oil phase (Sakellari et al., 2023). In this work, the aqueous P188 concentration even before addition to the pre-emulsion used for the particle dispersion production, was a lot lower than its critical micelle concentration (CMC, 17.9 mM) (Alexandridis et al., 1994). Therefore, the lack of micelles present in the systems to facilitate any mass transfer, in combination with the usage of a lipid (C888) with very low aqueous solubility (Samtlebe et al., 2012) has led to no losses compared to the already minimal crystalline matter reduction reported for the C888/T80 particles. Lastly, the fewer particle/oil contact points should be accounted, as the proportion of C888/P188 particles required to cover the oil droplet surface area was a lot less than that of the C888/T80 particles, due to the significantly larger emulsion droplet sizes produced with the former, as primarily shown for the NLCs (Figure 4).

**Figure 6.**
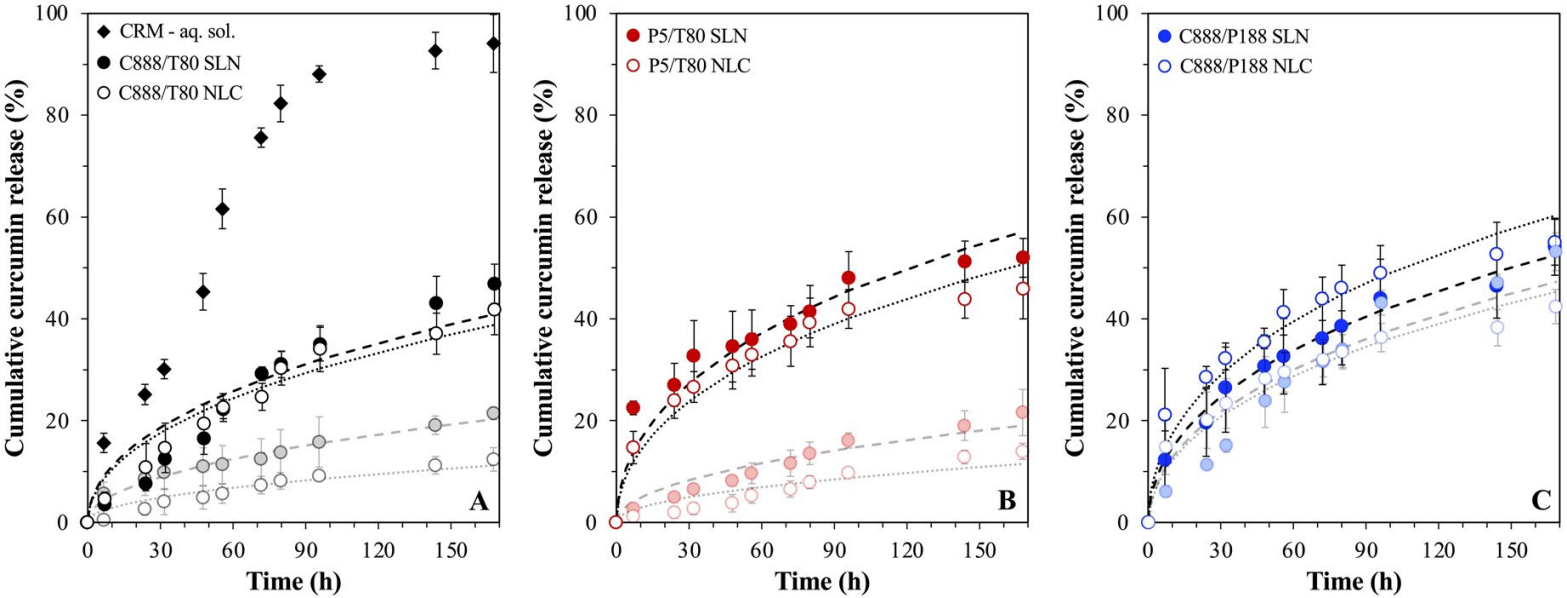
*In vitro* release profile of curcumin-loaded SLN- and NLC-stabilized emulsions formulated with either Tween^®^ 80 (T80) as surfactant and different types of solid lipid (A) Compritol^®^ 888 ATO (C888) and (B) Precirol^®^ ATO 5 (P5), or different surface active species (C) Poloxamer 188 (P188) and C888 as solid lipid. NLCs were fabricated with Miglyol^®^ 812 as the liquid lipid, at 30% w/w of the total lipid phase mass. The *in vitro* release kinetic Crank model (Eq. (3)) fitting of curcumin for each emulsion system stabilized by SLNs (dashed line) or NLCs (dotted line) is also presented. The release profile from a curcumin solution obtained under the same conditions (a) and that of the particles within the dispersion systems are also depicted in each respective graph. Graph A has been previously shown in the literature (Sakellari et al., 2023) and is provided here for comparison purposes.

With regard to their release regulation ability, both particle types prepared with P5 exhibited faster release compared to the particles fabricated with C888, while P5 NLCs release slightly less of their content than the P5 SLNs (Figure 6(A and B)). The greater loss of crystalline matter in the P5/T80 SLNs/NLCs (Figure 5) could account for the higher release rate, particularly in the first 48 h of the experiment. Conversely, the CRM release rate from both P188-formed SLNs and NLCs at the emulsion interface was only marginally higher to that exhibited by the particles in an aqueous dispersion setting (Figure 6(C)). The absence of any micelles available to facilitate the dissolution of solid matter and thereby release of any CRM associated with it, as well as the higher percentage of unadsorbed C888/P188 particles in the continuous phase (due to the lower number required to cover the smaller droplet surface area), were hypothesized to be the reasons for this lack of release profile changes. The calculated diffusion coefficient values were in the same order of magnitude for all particle-stabilized emulsions (Table 1), with only exception the P5/T80 SLNs that were characterized by a significantly higher *D* value, which aligns well with the fact that this was the particle type with the higher loss of crystalline matter. The EE and LC remained the same as for the dispersions (99.9 ± 0.0% and 0.5 ± 0.0%, respectively).

### 3.2. Release from lipid particle-stabilized emulsion droplets

Within this work, apart from their active carrying and release regulation ability, lipid particles acted also in tandem as Pickering emulsion stabilizers. The latter functionality contributes to two main aspects, with lipid particle-laden interfaces: (*i*) promoting/ensuring droplet stabilization; and (*ii*) acting as a barrier/regulator for the release of a secondary active encapsulated within the emulsion droplets. There are two rate-limiting steps relevant to the release of an active from within emulsion droplets; active diffusion within the oil droplet and toward the interface, and active transfer across the interfacial barrier (Washington & Evans, 1995; Kurukji et al., 2016; Spyropoulos et al., 2020; Sakellari et al., 2021b). For the systems studied here, it was shown (see ­section 2.10) that release was governed by the interfacial transfer of the active and thus the formulation properties of the lipid particles formulated here were expected to have an impact on this interfacial barrier. In order to test this hypothesis, a range of SLNs and NLCs (investigated earlier for their curcumin (primary model active) discharge regulation), were utilized (in this case) as blank colloidal species providing Pickering stabilization to emulsion droplets loaded with cinnamaldehyde, used as a (secondary) model hydrophobic active.

#### 3.2.1. The effect of lipid particle formulation

**Figure 7.**
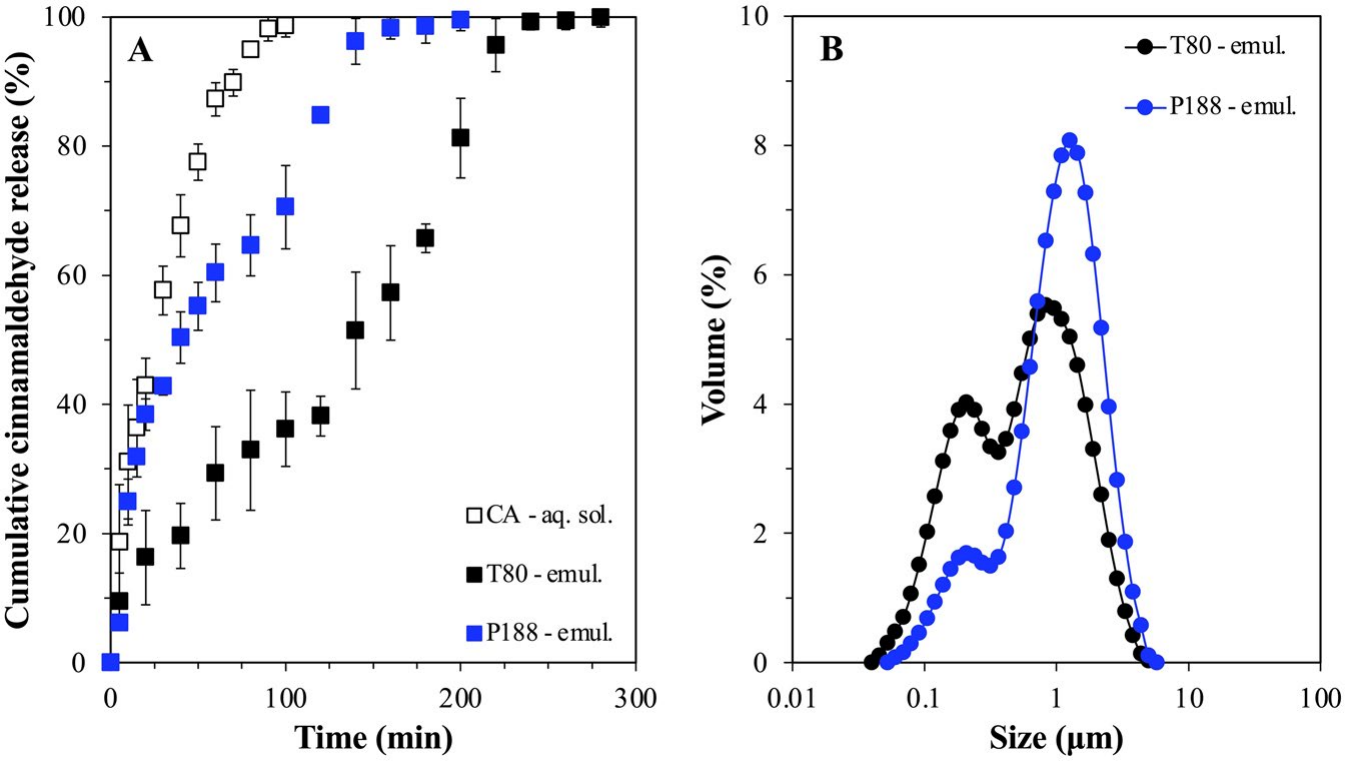
*In vitro* release profile of cinnamaldehyde-loaded emulsions stabilized with either Tween® 80 (T80) or Poloxamer 188 (P188) (a), droplet size distribution of the same systems (B). The release profile from a CA solution obtained under the same conditions is also depicted (a).

**Figure 8.**
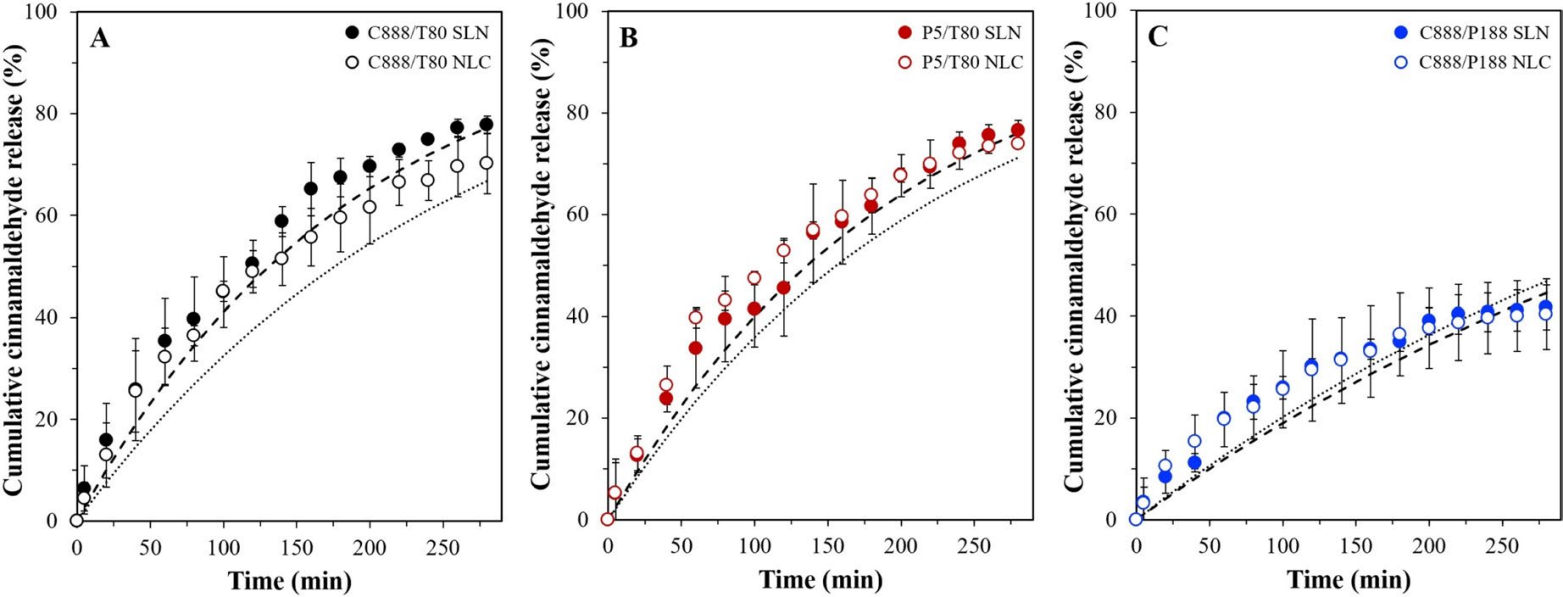
*In vitro* release profile of cinnamaldehyde-loaded emulsions stabilized by SLNs and NLCs formulated with either Tween^®^ 80 (T80) as surfactant and different types of solid lipid (A) Compritol^®^ 888 ATO (C888) and (B) Precirol^®^ ATO 5 (P5), or different surface active species (C) Poloxamer 188 (P188) and C888 as solid lipid. NLCs were fabricated with Miglyol^®^ 812 as the liquid lipid, at 30% w/w of the total lipid phase mass. The interfacial barrier-limited model (Eq. (6)) fits to the data for the release of cinnamaldehyde from the SLN- (dashed line) and NLC- (dotted line) stabilized emulsions are also presented.

For the purpose of establishing a reference for the impact of the particles’ interfacial presence on both the droplets’ active carrying and release capacity, simple emulsions fabricated with either of the two surface active species used for lipid particle preparation (T80 or P188) were also studied. Compared to a cinnamaldehyde (CA) solution, confining the active within the emulsion droplets appeared to slow down the release rate to a certain degree, with full discharge achieved within 100 min for the former (CA solution) and a delay (to full release) of 50 and 150 min recorded for the P188 and T80 stabilized emulsions, respectively (Figure 7(A)). Droplet size characteristics and EE/LC values were also determined, with both emulsions showing bimodal size distributions with a main peak at around 1 μm and a smaller one at ∼0.25 μm (Figure 7(B)), and no significant differences in the attained EE and LC values (approximately 83% and 7.6 × 10^−3^%, respectively) (Table 3). In contrast, the interfacial presence of the lipid particulates seemed to slow down the cinnamaldehyde release rate and overall percentage discharged. Approximately, 75% release was reached at 280 min for both C888/T80 and P5/T80 particles, and 40% was released at the same timescale for the C888/P188 counterparts, while almost identical profiles were obtained for SLNs and NLCs with the same formulation characteristics (Figure 8). In terms of the capacity of the particles to improve the amount of active remaining contained within the droplets, the EE and LC values were in the same range as for the simple emulsions (Table 3). This suggested that the active retention within the droplets was predominantly governed by the characteristics of the oil phase, rather than their interfacial decoration.

**Table 3.** Encapsulation efficiency (EE), loading capacity (LC), interfacial rate constant coefficient (*k*_1_), and coefficient of determination (*R*^2^) describing the model fitting for the interfacial barrier-limiting model of the cinnamaldehyde release data from emulsion systems. Identical lowercase letters indicate no significant differences between samples.

Formulation	EE (%)	LC × 10^−3^ (%)	*k*_1_ × 10^−15^ (cm^2^ s^−1^)	*R* ^2^
T80	84.2 ± 1.0^a^	7.8 ± 0.0^a^	–	–
P188	83.3 ± 2.8^a^	7.6 ± 0.0^b^	–	–
C888/T80 SLN	80.7 ± 1.1^a^	7.7 ± 0.0^c^	3.9 ± 1.5^a^	0.98
C888/T80 NLC	88.1 ± 3.8^a^	8.6 ± 0.0^d^	2.6 ± 0.4^a^	0.99
P5/T80 SLN	82.9 ± 2.5^a^	7.7 ± 0.0^e^	2.8 ± 0.6^a^	0.99
P5/T80 NLC	83.5 ± 3.3^a^	7.9 ± 0.0^f^	2.4 ± 0.5^a^	0.99
C888/P188 SLN	84.4 ± 1.4^a^	8.1 ± 0.0^g^	1900 ± 45^b^	0.94
C888/P188 NLC	88.0 ± 2.1^a^	8.1 ± 0.0^h^	1700 ± 53^c^	0.94
C888/T80 SLN − 64 °C 5 min	80.7 ± 0.9^a^	7.7 ± 0.0^c^	1.4 ± 0.7^a^	0.98
C888/T80 SLN − 64 °C 20 min	80.9 ± 0.8^a^	7.7 ± 0.0^c^	1.4 ± 0.9^a^	0.96
C888/T80 SLN − 64 °C 60 min	81.0 ± 1.0^a^	7.7 ± 0.0^c^	2.2 ± 0.5^a^	0.94

Even though o/w emulsions represent a suitable and adaptable platform for the encapsulation and delivery of poorly water-soluble actives, the absence of a more robust barrier at the droplet interface can lead to burst release and active expulsion overtime (Simovic & Prestidge, 2007; Frelichowska et al., 2009a,b; Dickinson, 2010). Herein, it was shown that differences in the interfacial composition, from surfactant molecules to lipid particles, and even more specifically changes in the structure of the particles themselves, did in fact alter the release kinetics. In literature, complete release from simple emulsions has been shown to occur over varying timescales ranging from a couple of minutes for small hydrophobic solutes such as chlorpromazine from soya oil droplets stabilized with P188 (Washington & Evans, 1995), to a couple of hours for the release of dibutylpthalte from bare polydimethylsiloxane droplets (Prestidge & Simovic, 2006). This aligns well with the data reported here, as any small difference could be related to disparities in the droplet sizes variations in the partition coefficient values between the used active, oil phase and dissolution medium, and/or altered release measurement methods. Concerning the effect of the particle addition, preliminary studies revealed that the incorporation of cinnamaldehyde did not alter the physical properties of the formed Pickering emulsions. Therefore, required information regarding the droplet sizes to estimate the interfacial rate constant (*k*_1_) values (Eq. (6)), was used according to Figure 4. The *k*_1_ values estimated in this work were in the range of 10^−15^ cm^2^s^−1^ for the emulsions stabilized by T80-formed particles and almost three orders of magnitude larger for the C888/P188 SLNs/NLCs-stabilized emulsions (Table 3). Similar values to the C888/P188 particle-stabilized emulsions have been described for dimethyl phthalate releasing from emulsions stabilized with SLNs prepared using whey protein isolate (3.1 × 10^−12^ cm^2^ s^−1^) (Sakellari et al., 2021b), as well as emulsions stabilized by surfactants with Pickering-like characteristics (Spyropoulos et al., 2020). On the contrary, emulsions stabilized with T80 particles gave values akin to previously reported data for emulsions stabilized by silica particles (Prestidge & Simovic, 2006; Simovic & Prestidge, 2007) and protein/polysaccharide co-precipitates (Kurukji et al., 2016). Such deviations could be ascribed to disparities in the active partition coefficient and dissolution medium solubility values amongst the various studies. However, despite the much greater *k*_1_ values for the C888/P188 SLNs/NLCs, the experimentally recorded release rate for CA was much slower compared to that of their C888/T80 and P5/T80 counterparts. This could be due to the fact that the interfacial area of the C888/P188 SLNs/NLCs-stabilized emulsions is much lower (almost 10-times higher droplet size) compared to the C888/T80 or P5/T80-stabilized droplets, which could be also contributing to the worse fitting of the interfacial-barrier model (Table 3).

Overall, both SLNs and NLCs were shown to act as effective interfacial barriers against the burst release of a model hydrophobic active enclosed in the lipid particle-stabilized emulsion droplets. What is more, it was demonstrated that lipid particle characteristics (in this case, the type of surfactant used during particle fabrication) can be significant factors in terms of active release across a lipid particle–laden interface. Thus, such particle characteristics could be controlled at the particle fabrication stage, in order to provide interfacial barriers with a specific release performance (Deshmukh et al., 2015; Ming et al., 2022). The same principle should potentially also apply to manipulating particle size, a feature that was not specifically investigated here and was practically kept unchanged across the studied SLN/NLC particles.

#### 3.2.2. The effect of interfacial sintering

**Figure 9.**
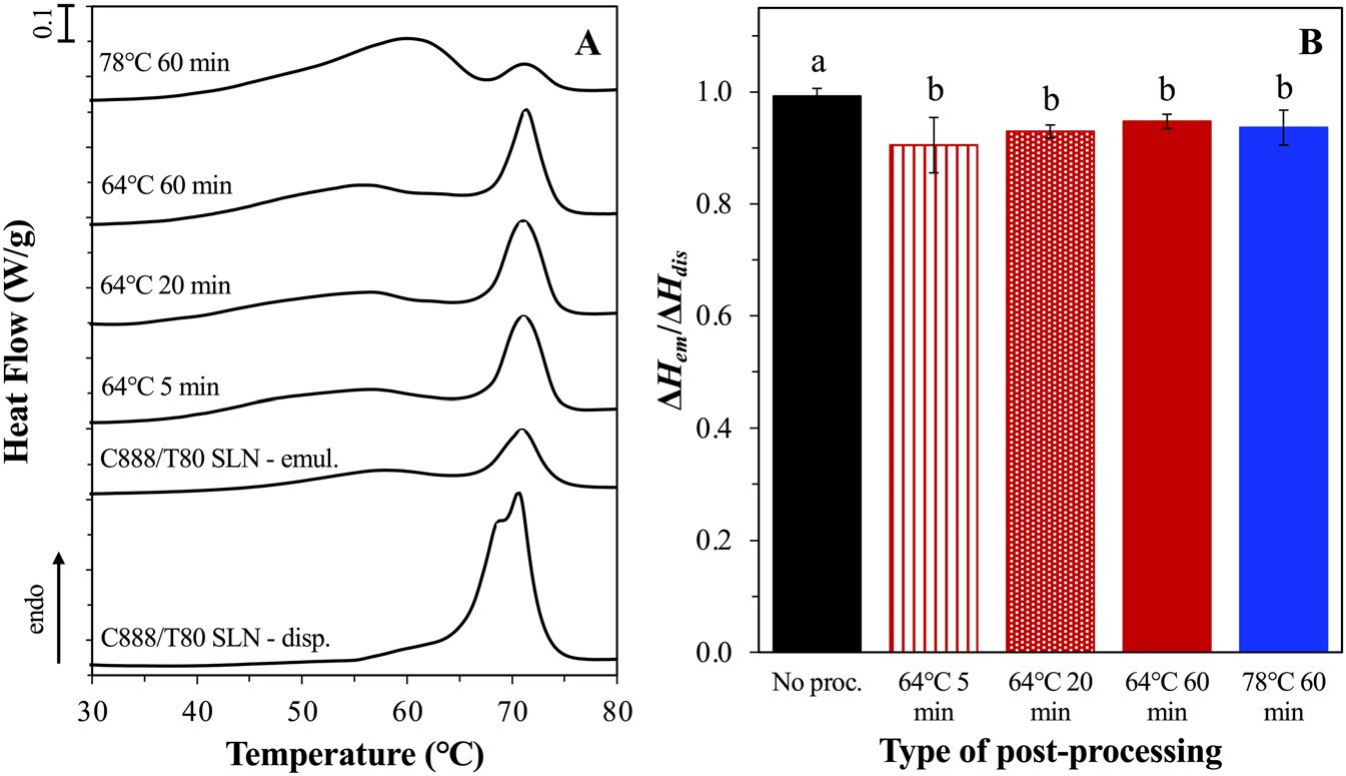
DSC melting thermograms of C888/T80 SLN-stabilized emulsions before and after sintering at 64 and 78 °C for varying durations (a). The curves were normalized for the amount of solid matter present in each sample and shifted along the ordinate for better visualization. The melting curve of the C888/T80 SLN dispersion is also provided for comparison purposes. Ratio of the melting enthalpies (Δ*H^T^_em_*/Δ*H_dis_*) of the emulsion systems presented in graph (a), representing the amount of crystalline material remaining within the emulsions post-processing (B). Identical lowercase letters indicate no significant differences between samples.

To further evaluate the plausibility of controlling the active discharge from within the o/w emulsion droplets by manipulating the lipid particle–laden interfacial architecture, the newly prepared Pickering emulsions were subjected (post-production) to thermal processing. The occurrence of solid bridge formation between neighboring fat crystals that are driven by mutual adhesion, also known as sintering, has been previously discussed in literature as a means of controlling the strength of a lipid-based structure and the texture of the resulting products (Johansson & Bergenståhl, 1995). Thermal sintering has been widely utilized as a preparation method for colloidosomes, thereby creating a robust layer at the oil/water interface that can provide not only improved protection against destabilization phenomena, but also create microcapsules suitable for carrying and delivering active molecules (Dinsmore et al., 2002; Yow & Routh, 2009). To this end, C888/T80 SLN-stabilized emulsions were heated (post-production) at either 64 °C or 78 °C under mild stirring for varying times, and changes in their physical properties and (release) performance were studied. According to earlier work using the particle-stabilized emulsions (Sakellari et al., 2023), the two peaks at ∼60 °C and 70 °C observed in their melting thermograms were ascribed to either particles associated with the emulsion interface (adsorbed) or particles remaining free in the continuous phase (unadsorbed), respectively. Therefore, the temperatures selected for this proof-of-concept sintering assessment were based on said melting events, presented in Figure 9(A).

**Figure 10.**
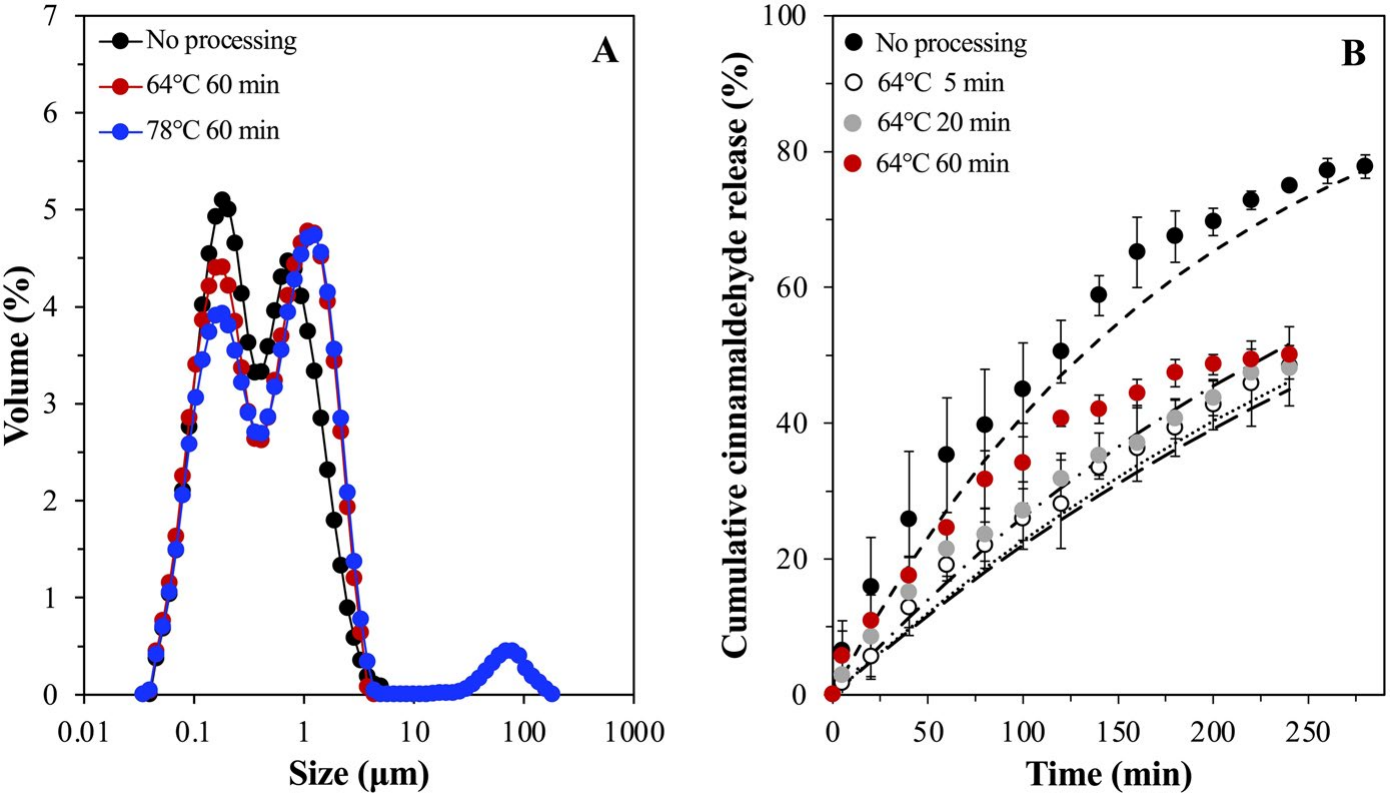
Droplet size distribution of C888/T80 SLN-stabilized emulsions before and after thermal processing at 64 and 78 °C for 60 min (a). *In vitro* release profile of cinnamaldehyde-loaded emulsions stabilized by C888/T80-SLNs after being subjected to thermal processing at 64 °C for varying durations (B). The profile of the respective emulsion system prior-sintering is also presented for comparison purposes. The interfacial barrier-limited model (Eq. (6)) fits to the data for the release of cinnamaldehyde from each emulsion system is also presented (­ ­ no processing, ···· 64 °C 5 min,—64 °C 20 min, –⋅ 64 °C 60 min).

Heating of the SLN-stabilized emulsions (post-production) at 64 °C for increasing timescales (5, 20 and 60 min) caused a statistically significant loss of crystalline matter compared to the untreated system, although the loss was overall minimal with all systems maintaining >90% of their initial solid content intact post thermal processing (Figure 9(B)). When the emulsions were heated at the highest temperature (78 °C), the melting profile of the system in terms of the relative intensity of the peaks appeared altered, with the peak at 62 °C being more pronounced than that at 70 °C, suggesting a decrease in the proportion of particles remaining unadsorbed in the continuous phase. Following the extended heating at 78 °C, partial phase separation was observed with a visible oil layer formed at the top of the emulsion. An increase in droplet size was also recorded here, possibly due to droplet and/or particle aggregation events induced by the complete melting of the lipid particles both at the droplet interface and within the continuous phase (Figure 10(A)). However, it is not clear whether shorter periods of exposure at this temperature would yield similarly compromised formulations. It is worth noting that despite the slight loss to their crystalline integrity, SLN-stabilized emulsions heated at 64 °C were able to maintain an unchanged droplet size distribution regardless of the duration of the thermal treatment.

In terms of release performance, emulsion that were thermally processed at 64 °C displayed a more sustained release profile (compared to their untreated predecessor) with no differences observed between them, and around 50% of CA being released within 240 min, as opposed to the 75% achieved by their parent (non-heated) formulation (Figure 10(B)). Fitting of the interfacial barrier-limiting model gave *k*_1_ values lower than for the unprocessed emulsion (Table 3). The release performance of samples heated at 78 °C was not further assessed due to the oil phase separation and discrepancies that this would cause to the calculation of the percentage of active discharging from the remaining physically intact droplets. In a study by Yow & Routh (2009), the formation of colloidosomes from colloidal poly(styrene-co-butyl acrylate) particles via sintering at varying temperatures and durations, was examined in relation to the release of a model active (fluorescein). It was shown that manipulation of the time and temperature provides control over the porosity and roughness of the formed colloidosome shell, with smoother shells providing longer release times. The importance of the sintering conditions when it comes to the tightness and durability of Pickering emulsion-based colloidosomes was also highlighted in another work (Yin et al., 2017), whereby reduced oil leakage was recorded for tightly packed colloidosome layers at the interface. Correspondingly, Rao et al. (2009) demonstrated that longer sintering (3 h) of C888 matrices at 80 °C led to retarded release rate of ketorolac tromethamine, owing to the increased extent and firmness of the sintered structure.

Overall, the results presented here suggest that a level of particle sintering was attained following the thermal processing of the Pickering emulsions, although further work is required to better elucidate the effect of the chosen conditions. Amongst these could be the influence of the occurring interactions and type of bridges formed depending on the composition of the interfacial layer (i.e. co-existence or not of particles and surfactant molecules) (Johansson & Bergenståhl 1995). Additionally, a more detailed study on the combined effects of temperature and duration of the heating/sintering step could be carried out, particularly on shorter exposure periods to temperatures above the solid lipids’ melting temperature, that could potentially inform on the microstructural integrity of the formulation at critical conditions.

### 3.3. Co-release from lipid particle-stabilized emulsions

**Figure 11.**
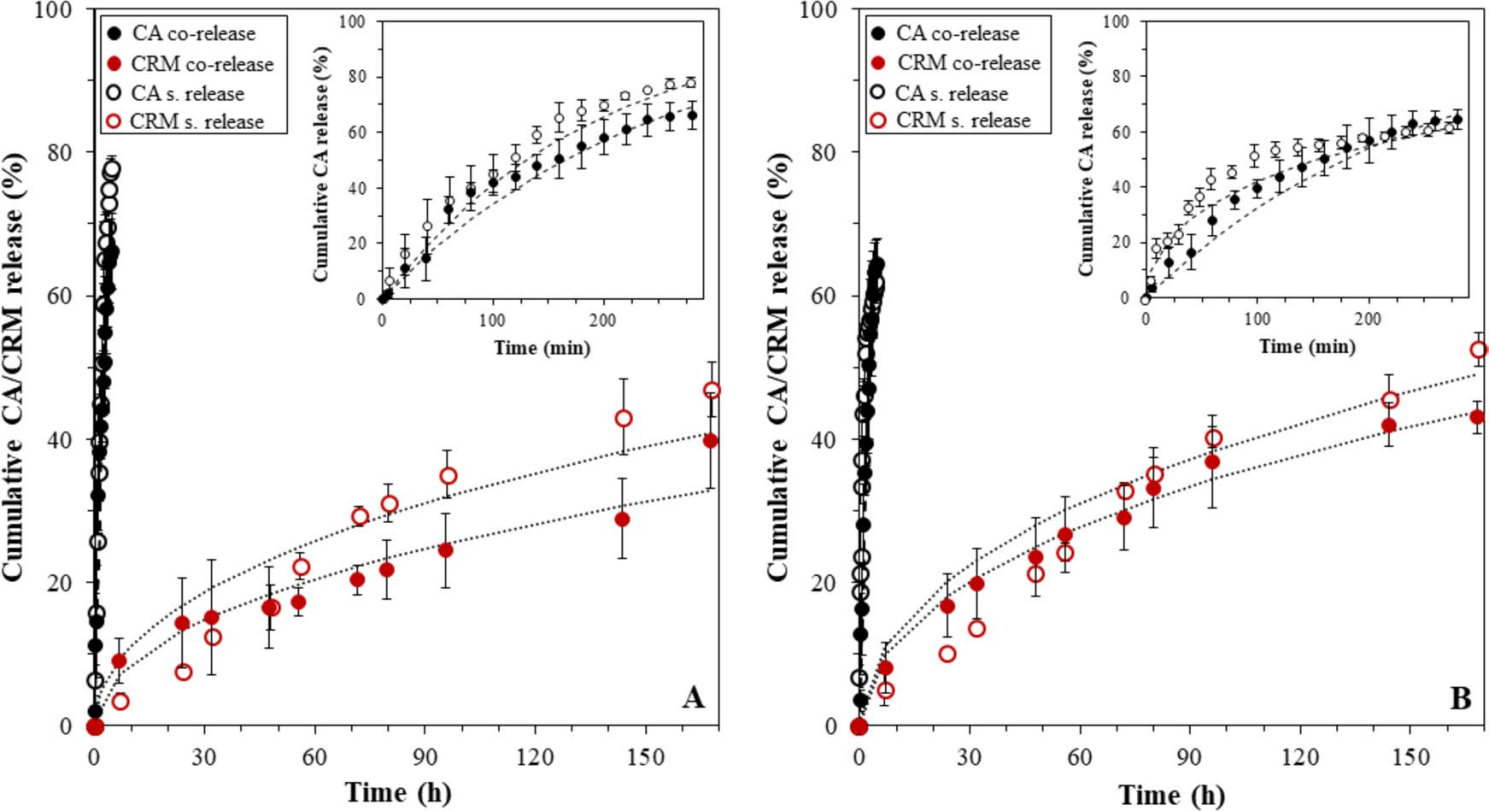
*In vitro* co-release profiles of cinnamaldehyde-loaded emulsion droplets stabilized by curcumin-loaded C888/T80-SLNs measured immediately after preparation (A) and after 1 month of emulsion storage (B). Data are presented at longer (main graph) and shorter (inset graph) timescales to demonstrate differences in the release rates. The Crank model (Eq. (3)) fitting for all curcumin data (dotted lines) and the interfacial barrier-limited model (Eq. (6)) fitting for all cinnamaldehyde curves (dashed lines) are also presented. For comparison purposes, the single release profiles of curcumin-loaded C888/T80-SLN-stabilized (blank) emulsions and CA-loaded emulsions stabilized by blank C888/T80 SLNs are included in both graphs.

Having established the capability of both lipid particles and lipid particle-stabilized droplets to separately act as effective carriers and delivery systems of model hydrophobic actives, this part of the work scrutinized whether these performances perpetuated when the lipid-particle stabilized emulsion was utilized as a co-delivery platform. Previous work using lipid-particle stabilized emulsions as a co-delivery formulation has provided evidence that this type of carrier platform could indeed facilitate the independent co-delivery of actives by separately measuring their release behavior (Sakellari et al., 2021b). Herein, the co-release performance of cinnamaldehyde-loaded emulsion droplets stabilized by curcumin-loaded C888/T80 SLNs, was assessed by simultaneously acquiring the release profiles of the two actives under the same experimental conditions (Figure 11).

The co-release profiles for both curcumin (releasing from the C888/T80 SLNs) and cinnamaldehyde (discharging from the o/w droplets) showed no significant differences to the profiles that were individually acquired (Figure 11(A)). As was previously recorded, the highest percentage of cinnamaldehyde released over 280 min, while curcumin showed a relatively more sustained release rate with approximately 40% being released over the 7-day assessment. The *D* for CRM and *k*_1_ constant for CA (130 × 10^−20^ cm^2^ s^−1^ and 3.2 × 10^−15^ cm^2^ s^−1^, respectively) were in accordance with what was reported above for the singly measured release rates (see Tables 1 and 3, respectively). More importantly, the co-release behavior measured immediately after formation, was preserved even after 1 month of emulsion storage, with no major disparities being observed in the profiles or release kinetics (*D*** **=** **250 × 10^−20^ cm^2^ s^−1^ and *k*_1_ = 4.4 × 10^−15^ cm^2^s^−1^) (Figure 11(B)). The co-encapsulation of the two actives did not alter the ability of either compartment in the formulation (i.e. lipid particles and emulsion droplets) to act as an independent active carrier, with no significant differences in the EE and LC values compared to the single encapsulation formulations.

## 4. Conclusions

The present study demonstrated the potential of SLN and NLC particles to simultaneously regulate the co-release performance of an active encapsulated within the particles themselves (acting as a carrier), but also that of a secondary active contained within the Pickering emulsion droplets (acting as a barrier). In terms of their identity as a carrier, investigation of the effect of changes to lipid particle formulation parameters, namely type of solid lipid and surfactant used, indicated that the release rate of the encapsulated active was predominantly governed by its relative location within the lipid matrix. Following confirmation of the Pickering stabilization capacity of the same particles, changes to their release control capability once within an emulsion setting were shown to be related to their formulation characteristics. The compatibility between the particles’ solid lipid and emulsion oil and the presence of surfactant micelles were suggested to have an inverse effect on the particles’ ability to maintain control over the discharge of their active. In terms of their identity as a barrier, all particles, regardless of their composition, exhibited improved control compared to surfactant decorated interfaces, although the permeability of the interfacial layer appeared to greatly affect the release rate. In view of the latter, a post-production thermal approach was adopted to demonstrate that interfacial sintering of the particles and hence creation of a less permeable layer could enhance their barrier capacity and further impede active release from the droplets. Lastly, the hypothesis that each constituent of this system could extend its performance from the single to a dual release (co-release) setting, even when such markedly different individual release profiles (in terms of timescales) were chosen, was corroborated.

Overall, the results presented here underlined the aptitude of the developed Pickering emulsion platforms as promising systems for the compartmentalized co-encapsulation and independently controlled co-delivery of two actives, owing to their tunable lipid particle-decorated interfaces. Such modulation potential was manifested by the ability to effectively adjust the release profiles individually at a single active level, and then essentially transcribe these into a dual delivery platform. In terms of utility within the drug delivery arena, combining individual release profiles of shorter timescales could cater to co-delivery via an oral administration route, while more prolonged delivery profiles could be combined to develop long-acting injectables (Wilkinson et al., 2022) with a co-delivery capacity.

## Data Availability

The data that support the findings of this study are available from the corresponding author, [GIS], upon reasonable request.
